# Assisted Reproductive Technology and Risk of Childhood Cancers

**DOI:** 10.1001/jamanetworkopen.2022.30157

**Published:** 2022-08-31

**Authors:** Shiue-Shan Weng, Yen-Tsung Huang, Yi-Ting Huang, Yi-Ping Li, Li-Yin Chien

**Affiliations:** 1 Institute of Public Health, National Yang Ming Chiao Tung University, Yang-Ming Campus, Taipei City, Taiwan; 2 Institute of Statistical Science, Academia Sinica, Taipei City, Taiwan; 3 Department of Obstetrics and Gynecology, Shin Kong Wu Ho-Su Memorial Hospital, Taipei City, Taiwan; 4 Institute of Community Health Care, National Yang Ming Chiao Tung University, Yang-Ming Campus, Taipei City, Taiwan

## Abstract

**Importance:**

The number of children born through the use of assisted reproductive technology (ART) has been increasing. These children may have higher risks for epigenetic alteration and adverse perinatal outcomes, which may be associated with childhood cancers.

**Objective:**

To determine the associations between different modes of conception and childhood cancers and potential mediation by preterm birth and low birth weight.

**Design, Setting, and Participants:**

This nationwide, population-based cohort study included registry data from 2 308 016 eligible parents-child triads in Taiwan from January 1, 2004, to December 31, 2017. A total of 1880 children with incident childhood cancer were identified. Data were analyzed between September 1, 2020, and June 30, 2022.

**Exposure:**

Mode of conception, defined as (1) natural conception, (2) subfertility and non-ART (ie, infertility diagnosis but no ART-facilitated conception), or (3) ART (ie, infertility diagnosis and ART-facilitated conception).

**Main Outcomes and Measures:**

Diagnosis of childhood cancer according to the *International Classification of Childhood Cancers, Third Edition*.

**Results:**

The mean (SD) paternal and maternal ages were 33.28 (5.07) and 30.83 (4.56) years, respectively. Of the 2 308 016 children, 52.06% were boys, 8.16% were born preterm, and 7.38% had low birth weight. During 14.9 million person-years of follow-up (median, 6 years [IQR, 3-10 years]), ART conception was associated with an increased risk of any type of childhood cancers compared with natural conception (hazard ratio, 1.58; 95% CI, 1.17-2.12) and subfertility with non-ART conception (hazard ratio, 1.42; 95% CI, 1.04-1.95). The increased cancer risk of children conceived with ART was mainly owing to leukemia and hepatic tumor. The increased cancer risk associated with ART conception was not mediated by preterm birth or low birth weight.

**Conclusions and Relevance:**

In this cohort study, children conceived via ART had a higher risk of childhood cancers than those conceived naturally and those born to parents with an infertility diagnosis did not use ART. The increased risk could not be explained by preterm birth or low birth weight.

## Key Points

**Question:**

Are children conceived via assisted reproductive technology (ART) at a higher risk of childhood cancers?

**Findings:**

In this nationwide population-based cohort study of 2 308 016 parents-child triads in Taiwan, children conceived via ART were at a statistically significant increased risk for childhood cancers compared with children conceived naturally and those born to parents with an infertility diagnosis who did not use ART. This association was not mediated by preterm birth and low birth weight.

**Meaning:**

The findings suggest that ART conception is associated with an increased risk for childhood cancers, and the increased risk cannot be attributed to preterm birth or low birth weight.

## Teaser

This cohort study examines the risk of childhood cancers among children conceived by assisted reproductive technology in Taiwan.

## Introduction

The number of infants born via assisted reproductive technology (ART) has increased worldwide.^1^ In Taiwan in 2018, approximately 1 in every 20 neonates was born with the help of ART (5.7% of all births).^2,3^ However, children born to parents with fertility problems or undergoing infertility treatment have been found to be at increased risk of epigenetic alterations^4,5^ and adverse perinatal outcomes,^6,7,8^ possibly associated with childhood cancers.^9,10,11,12,13,14^

Current evidence on the association between ART conception and childhood cancers remains debatable. Some studies indicated that ART conception increases the risk of childhood cancers,^15,16,17,18,19^ but others found no association.^20,21,22,23^ This discrepancy may be due to several factors. First, different reference groups were used in these studies, and very few studies have differentiated children of parents who had an infertility diagnosis with or without fertility treatments from those who delivered after natural conception to provide an appropriate comparison.^16,19,22^ Male infertility also has rarely been considered as an exposure variable. Second, only a few studies adjusted for potential confounders or adjusted for very few confounders.^15,16,17,19,20,21,22,23^ In circumstances in which both exposure and outcome are rare events, few studies used a nationwide or large population-based design to reduce selection bias and avoid the limited number of exposed cases.^15,17,18,21^ Finally, to our knowledge, no previous study explored whether perinatal outcomes mediate the association between mode of conception and childhood cancers.^24^

Therefore, we designed a population-based cohort study using nationwide registry data from Taiwan between 2004 and 2017 to examine the association of mode of conception (natural conception, subfertility and non-ART, and ART) with the risk of childhood cancers. We also assessed the potential mediating role of perinatal outcomes in this association.

## Methods

### Data Sources

Data between January 1, 2004, and December 31, 2017, were retrieved from the Maternal and Child Health Database, which contains 99.78% of all parents-child triads (defined as 1 offspring and both parents) in Taiwan,^25^ and its linkages to 6 national administrative databases. These national registry databases have previously been evaluated as valid and complete.^26,27,28,29^ The detailed linkage process across databases and how to assess these databases are described in eFigure 1 and the eAppendix in the Supplement.

The institutional review board of National Yang-Ming University approved the study and waived the requirement for informed consent for deidentified data. This study followed the Strengthening the Reporting of Observational Studies in Epidemiology (STROBE) reporting guideline.

### Cohort Identification and Exposure Assessment

Among the successfully matched parents-child triads (n = 2 553 583) from the national administrative databases, we excluded those with (1) parental age younger than 20 years; (2) a parent with a recorded addiction to alcohol, tobacco, or drugs during pregnancy or a history of cancer; (3) conception using sperm or oocyte donations; (4) an older sibling who was born in the same year as the index child; and (5) a parent of a foreign origin (owing to a lack of medical records before coming to Taiwan) (eFigure 3 in the Supplement).

Three groups of children were classified based on mode of conception: (1) natural conception, (2) subfertility and non-ART, and (3) ART. For classification as ART, all of the following 3 criteria had to be met. First, one of the parents had to have an infertility diagnosis (female: *International Classification of Diseases, Ninth Revision *[*ICD-9*] codes 628.0-628.9 or *International Statistical Classification of Diseases and Related Health Problems, Tenth Revision *[*ICD-10*] codes N97.0-N97.9; male: *ICD-9* 606 or *ICD-10* N46), because an infertility diagnosis is a prerequisite for ART in Taiwan.^30^ Second, a clinical pregnancy had to be recorded following ART (fresh or frozen embryo). Third, to minimize misclassification and to identify which child was born following ART if there were more than 1 child in a household, the offspring’s birth date had to be within 290 days after the date of transfer (approximately 44 weeks’ gestational age) (eFigure 2 in Supplement). Subfertility and non-ART was determined if one of the parents had an infertility diagnosis and no history of ART or record of clinical pregnancy following ART. Natural conception was determined if the parents had neither an infertility diagnosis nor ART. The 3 groups were tracked from birth until they developed childhood cancers, met any of the exclusion criteria, died during the observation period, or reached the end of the follow-up period (December 31, 2017), whichever came first.

### Definitions of Childhood Cancers

Cancers that occurred between birth and the end of the follow-up (offspring age ranging from birth to 13 years) were identified as cases. Information on cancer diagnosis was from the linkage to the Taiwan Cancer Registry, which records all incident cancer cases. This database has high completeness and accuracy.^27^ Childhood cancers were categorized using the *International Classification of Childhood Cancers, Third Edition *(*ICCC-3*),^31^ based on morphology and topography codes from the *International Classification of Diseases for Oncology, Third Edition* (*ICD-O-3*) (eTable 1 in Supplement).

### Potential Confounders and Mediators

Several confounders were taken into account based on variables that are associated with modes of conception and can affect childhood cancers: maternal age^32,33^; paternal age^32,34^; maternal addiction to tobacco, alcohol, or drugs^35,36,37^; parity^14,16^; abortion history (*ICD-9-CM*: 630-639; *ICD-10-CM*: O00-O08)^38,39^; socioeconomic status^38,40^; parental cancer history; and residential urbanization level.^41^ A proxy for parental socioeconomic status was household income at birth, calculated by the National Health Insurance program, which covers 99.6% of Taiwan’s residents.^42^ Residential urbanization level was based on an urban-rural classification of townships in Taiwan.^43^ Preterm birth and low birth weight were reported to be associated with the conception mode and the frequency of childhood cancers.^6,7,8,11,12,13,14^ Therefore, these variables were examined for whether they met the criteria of potential mediators. The criteria for a potential mediator were as follows: (1) a change in the level of the exposure variable significantly affects that of the mediator, and (2) a change in the level of the mediator significantly affects that of the outcome.^44^ Only the variables that met the criteria for a potential mediator were included in the mediation analysis.

### Statistical Analysis

Statistical analyses were conducted between September 1, 2020, and June 30, 2022. The person-years at risk, crude incidence rates, and incidence rate difference of childhood cancers were calculated. The association between the mode of conception and childhood cancers was examined using Cox proportional hazards models to estimate hazard ratios (HRs) after adjustment for maternal age, paternal age, child’s birth year, child’s sex, parity, household income at birth, residential urbanization level, and abortion history. We further stratified the association between ART conception and childhood cancers by embryo type (fresh or frozen), source of infertility (paternal, maternal, or both), and child’s sex.

The primary analysis of the association between the conception mode and childhood cancers comprised offspring who were the first to have childhood cancer in their household to avoid correlation between siblings. It was noted that only 4 children (0.2%) were the second to have childhood cancer in their families, and we obtained similar estimates when they were included in the analysis. Furthermore, the primary results used complete case analysis because the missing data on adjusted variables were very few (1.79%). The complete case result was similar to that obtained using the multivariate imputation by chained equations method to deal with missing data.^45,46^ The proportional hazards assumption of the Cox model was examined graphically based on scaled Schoenfeld residuals and showed no violation. An E-value analysis was performed to estimate how large an unmeasured confounder must be to render the observed association a null estimate.^47^

We used the 4-way decomposition using the med4way command in STATA, version 15.0 (StataCorp LLC) to perform mediation analysis, allowing for the setting of binary mediator and survival outcome under a rare outcome assumption.^48,49^ This method decomposes the association between conception mode and childhood cancers (total association) into 4 components: (1) controlled direct association (independent of the mediator and interaction between ART and the mediator), (2) pure indirect association (mediation only), (3) reference interaction (interaction only between ART and the mediator), and (4) mediated interaction (mediation and interaction between ART and the mediator). Estimates were reported as excess relative risks after adjusting for the confounders.

All statistical analyses were performed using STATA version 15.0.^50^ All *P* values were 2-tailed and were set at a critical significance level of 5%.

## Results

### Characteristics of Study Participants

After applying the exclusion criteria, the study cohort consisted of 2 308 016 parents-child triads (eFigure 3 in the Supplement), giving 14 926 318.5 person-years of observation during a median follow-up of 6 years (IQR, 3-10 years). The characteristics of study participants are presented in Table 1. Among 2 308 016 children (mean [SD] paternal age, 33.28 [5.07] years; mean [SD] maternal age, 30.83 [4.56] years), 1 201 585 (52.06%) were boys and 1 106 424 (47.94%) were girls; 2 236 562 (96.90%) were singletons; 188 231 (8.16%) were born preterm; and 170 312 (7.38%) had low birth weight. Children born after ART conception had the highest percentages of multiple gestations (23 081 [48.95%]), preterm birth (17 185 [36.45%]), and low birth weight (17 141 [36.35%]). Additionally, their parents were more likely to be older and primiparous, live in more urbanized areas, and have a higher household income.

**Table 1.** Characteristics of Study Participants

Characteristic	No. (%)			
	Total (N = 2 308 016)	Natural conception (n = 1 794 555)	Subfertility and non-ART conception (n = 466 309)	ART conception (n = 47 152)
Paternal age, mean (SD), y	33.28 (5.07)	32.89 (5.04)	34.42 (4.89)	37.24 (4.55)
Missing data	41 332 (1.79)	35 898 (2.00)	5279 (1.13)	155 (0.33)
Maternal age, mean (SD), y	30.83 (4.56)	30.39 (4.50)	32.10 (4.43)	34.89 (3.68)
Missing data	41 332 (1.79)	35 898 (2.00)	5279 (1.13)	155 (0.33)
Birth year				
2004-2007	663 801 (28.76)	522 975 (29.14)	133 028 (28.53)	7798 (16.54)
2008-2012	793 445 (34.38)	615 601 (34.30)	163 695 (35.10)	14 149 (30.01)
2013-2017	850 770 (36.86)	655 979 (36.55)	169 586 (36.37)	25 205 (53.45)
Sex				
Male	1 201 585 (52.06)	938 323 (52.29)	238 865 (51.74)	24 397 (51.22)
Female	1 106 424 (47.94)	856 227 (47.71)	227 442 (48.26)	22 755 (48.78)
Missing data	7 (<0.01)	NR	NR	NR
Parity				
1	1 533 591 (66.45)	1 180 938 (65.81)	311 827 (66.87)	40 826 (86.58)
≥2	774 425 (33.55)	613 617 (34.19)	154 482 (33.13)	6326 (13.42)
No. of births				
Singleton	2 236 562 (96.90)	1 766 157 (98.42)	446 334 (95.72)	24 071 (51.05)
Multiple gestations	71 454 (3.10)	28 398 (1.58)	19 975 (4.28)	23 081 (48.95)
Residential urbanization level				
Cluster 1 (highest)	663 775 (28.76)	512 765 (28.57)	134 106 (28.76)	16 904 (35.85)
Cluster 2	565 476 (24.50)	434 410 (24.21)	117 939 (25.29)	13 127 (27.84)
Cluster 3	541 244 (23.45)	423 539 (23.60)	108 303 (23.23)	9402 (19.94)
Clusters 4-7 (lowest)^a^	537 512 (23.29)	423 834 (23.62)	105 959 (22.72)	7719 (16.37)
Missing data	9 (<0.01)	NR	NR	0
Household income quartile at time of birth				
Q1 (lowest)	549 566 (23.81)	445 180 (24.81)	98 403 (21.10)	5983 (12.69)
Q2	593 643 (25.72)	464 459 (25.88)	117 893 (25.28)	11 291 (23.95)
Q3	589 151 (25.53)	462 905 (25.79)	116 446 (24.97)	9800 (20.78)
Q4 (highest)	575 656 (24.94)	422 011 (23.52)	133 567 (28.64)	20 078 (42.58)
Gestational age, wk				
Median (IQR)	38 (38-39)	38 (38-39)	38 (37-39)	37 (36-38)
<37	188 231 (8.16)	125 996 (7.02)	45 050 (9.66)	17 185 (36.45)
37-42	1 967 502 (85.25)	1 551 716 (86.47)	390 434 (83.73)	25 352 (53.77)
>42	142 (0.01)	123 (0.01)	19 (0.00)	0
Missing data	152 141 (6.59)	116 720 (6.50)	30 806 (6.61)	4615 (9.79)
Birth weight, g				
Median (IQR)	3090 (2820-3350)	3100 (2840-3350)	3080 (2800-3350)	2650 (2242-3074)
<2500	170 312 (7.38)	113 675 (6.33)	39 496 (8.47)	17 141 (36.35)
2500-4200	1 974 629 (85.56)	1 555 803 (86.70)	393 524 (84.39)	25 302 (53.66)
>4200	10 934 (0.47)	8357 (0.47)	2483 (0.53)	94 (0.20)
Missing data	152 141 (6.59)	116 720 (6.50)	30 806 (6.61)	4615 (9.79)
Abortion				
No	1 298 627 (56.27)	1 039 989 (57.95)	231 620 (49.67)	27 018 (57.30)
Yes	1 009 389 (43.73)	754 566 (42.05)	234 689 (50.33)	20 134 (42.70)
Source of infertility				
Paternal	32 168 (6.26)	NA	31 749 (6.81)	419 (0.89)
Maternal	409 767 (79.80)	NA	379 158 (81.31)	30 609 (64.92)
Both	71 526 (13.93)	NA	55 402 (11.88)	16 124 (34.20)

Abbreviations: ART, assisted reproductive technology; NA, not applicable; NR, not reported due to the number being smaller than 3 to protect patient confidentiality under the Taiwan Data Protection Law.

^a^ Grouped cluster 4 to cluster 7 together owing to the small sample size in cells.

### Risk of Childhood Cancers

We identified 1880 offspring with incident childhood cancers. The incidence rates and incidence rate differences of childhood cancers by mode of conception are presented in Table 2 and Table 3. The incidence rates of childhood cancers per million person-years were the highest for the ART group, followed by subfertility and non-ART, and lowest for natural conception for any type of cancer (ART, 203.1; non-ART, 137.6; and natural, 121.4), leukemias (ART, 56.2; non-ART, 34.4; and natural, 29.8), and hepatic tumors (ART, 34.6; non-ART, 9.9; and natural, 8.2). The incidence rate of retinoblastoma was higher in the ART group (22.3) than the natural conception (5.6) and subfertility and non-ART (5.3) groups.

**Table 2.** Incidence Rates, Incidence Rate Difference, and Associations Between Mode of Conception and Childhood Cancers

Types of childhood cancer	No. of cases^a^			Incidence rate per million person-years			Incidence rate difference compared with natural conception per million person-years (95% CI)		HR (95% CI)^b^	
	Natural	Non-ART	ART	Natural	Non-ART	ART	Non-ART	ART	Non-ART	ART
Any type of childhood cancers	1417	416	47	121.4	137.6	203.1	16.2 (−1.5 to 30.8)	81.7 (23.3 to 140.1)	1.14 (0.99 to 1.27)	1.58 (1.17 to 2.12)
Leukemia	348	104	13	29.8	34.4	56.2	4.9 (−2.7 to 11.9)	26.4 (4.3 to 57.0)	1.19 (0.95 to 1.48)	2.10 (1.20 to 3.70)
Hepatic tumors	96	30	8	8.2	9.9	34.6	1.7 (−2.2 to 5.6)	26.3 (2.3 to 50.3)	1.09 (0.72 to 1.66)	2.71 (1.28 to 5.73)
Lymphomas and reticuloendothelial neoplasms	73	15	<5	6.3	4.3	5	−1.3 (−4.2 to 1.6)	−1.9 (−10.5 to 6.7)	0.87 (0.50 to 1.52)	0.94 (0.13 to 6.85)
CNS and miscellaneous intracranial and intraspinal neoplasms	237	86	5	20.3	28.4	21.6	8.1 (−1.6 to 14.7)	1.3 (−1.8 to 20.4)	1.42 (0.98 to 1.84)	1.16 (0.47 to 2.84)
Neuroblastoma and other peripheral nervous cell tumors	187	55	<5	16	18.2	17.3	2.2 (−3.2 to 7.5)	1.3 (−15.8 to 18.4)	1.10 (0.81 to 1.50)	0.91 (0.33 to 2.47)
Retinoblastoma	101	33	5	8.7	10.9	22.3	2.3 (−1.8 to 6.4)	13.7 (−1.9 to 27.9)	1.22 (0.82 to 1.83)	2.57 (0.98 to 5.75)
Renal tumors	65	16	<5	5.6	5.3	12.9	−2.8 (−3.2 to 2.7)	7.4 (−7.3 to 22.1)	0.95 (0.55 to 1.67)	1.86 (0.57 to 6.11)
Malignant bone tumors	NR	NR	0	0.1	0.3	NA	0.3 (−0.4 to 0.9)	−0.1 (−0.3 to 0.1)	3.62 (0.21 to 63.11)	NA
Soft tissue and other extraosseous sarcomas	102	30	<5	8.7	9.9	4.3	1.2 (−2.8 to 5.1)	−4.4 (−13.1 to 4.2)	1.18 (0.78 to 1.79)	0.47 (0.07 to 3.41)
Germ cell tumors, trophoblastic tumors, and neoplasms of the gonads	170	36	<5	14.6	11.6	8.6	−3.0 (−7.4 to 1.4)	−5.9 (−1.8 to 6.3)	0.75 (0.52 to 1.10)	0.55 (0.14 to 2.25)
Other malignant epithelial neoplasms and malignant melanomas	NR	NR	<5	3.3	2.3	4.5	1.0 (−0.6 to 2.7)	1.1 (−1.3 to 3.1)	2.26 (0.81 to 6.33)	1.58 (0.56 to 4.36)

Abbreviations: ART, assisted reproductive technology; CNS, central nervous system; HR, hazard ratio; NA, not applicable; NR, not reported due to the number being smaller than 3 to protect patient confidentiality under the Taiwan Data Protection Law.

^a^ The first-occurring childhood cancer in their household.

^b^ Reference group was natural conception. Adjusted for maternal age, paternal age, child’s birth year, child’s sex, parity, socioeconomic status, population density of living areas, and abortion history.

**Table 3.** Comparison of Childhood Cancer Risk by ART Conception and Subfertility and Non-ART Conception

Types of childhood cancer	No. of cases^a^		Incidence rate difference per million person-years (95% CI)	HR (95% CI)^b^
	Non-ART	ART		
Any type of childhood cancers	416	47	65.5 (5.7 to 125.1)	1.42 (1.04 to 1.95)
Leukemia	104	13	21.8 (9.5 to 62.0)	1.88 (1.03 to 3.43)
Hepatic tumors	30	8	24.6 (4.3 to 48.9)	2.41 (1.05 to 5.52)
Lymphomas and reticuloendothelial neoplasms	15	<5	−6.4 (−9.5 to 8.2)	0.92 (0.12 to 7.35)
CNS and miscellaneous intracranial and intraspinal neoplasms	86	5	−6.8 (−26.7 to 13.0)	0.80 (0.32 to 2.01)
Neuroblastoma and other peripheral nervous cell tumors	55	<5	−9.0 (−18.5 to 16.7)	0.89 (0.31 to 2.53)
Retinoblastoma	33	5	11.4 (−1.3 to 7.00)	2.32 (0.98 to 5.28)
Renal tumors	16	<5	7.7 (−7.2 to 22.6)	1.82 (0.50 to 6.65)
Malignant bone tumors	NR	0	−0.3 (−1.0 to −0.3)	NA
Soft tissue and other extraosseous sarcomas	30	<5	−5.6 (−1.5 to 3.6)	0.46 (0.06 to 3.51)
Germ cell tumors, trophoblastic tumors, and neoplasms of the gonads	36	<5	−2.9 (−15.5 to 9.6)	0.69 (0.16 to 2.95)
Other malignant epithelial neoplasms and malignant melanomas	NR	<5	2.2 (−4.4 to 8.7)	1.83 (0.56 to 9.36)

Abbreviations: ART, assisted reproductive technology; CNS, central nervous system; HR, hazard ratio; NA, not applicable; NR, not reported owing to the number being smaller than 3 to protect patient confidentiality under the Taiwan Data Protection Law.

^a^ The first-occurred childhood cancer in their household.

^b^ Adjusted for maternal age, paternal age, child’s birth year, child’s sex, parity, socioeconomic status, population density of living areas, and abortion history.

After adjustment for confounders, ART conception was associated with an increased risk for any type of childhood cancers (HR, 1.58; 95% CI, 1.17-2.12), leukemias (HR, 2.10; 95% CI, 1.20-3.70), and hepatic tumors (HR, 2.71; 95% CI, 1.28-5.73) (Table 2) when compared with natural conception. When compared with subfertility and non-ART, ART was also associated with higher risks for any type of childhood cancers (HR, 1.42; 95% CI, 1.04-1.95), leukemias (HR, 1.88; 95% CI, 1.03-3.43), and hepatic tumors (HR, 2.41; 95% CI, 1.05-5.52) (Table 3). Other types of childhood cancers showed no association with mode of conception (Table 2 and Table 3). The risk of childhood cancers was not significantly different between subfertility and non-ART conception and natural conception groups.

Stratified analysis was performed to examine whether the above associations differed by source of infertility, embryo type (eTable 2 in the Supplement), and child sex (eTable 3 in the Supplement). The association between mode of conception and childhood cancer did not differ significantly by source of infertility and child sex. Use of frozen embryos was not associated with cancer risk, whereas use of fresh embryos was associated with increased cancer risk. Sensitivity analyses of unmeasured confounders for those associations indicated that fairly substantial confounding would be required to explain the associations (any childhood cancers: HR, 2.54; lower limit of 95% CI, 1.62; leukemias: OR, 3.62; lower limit of 95% CI, 1.69; hepatic tumors: OR, 4.86; lower limit of 95% CI, 1.88).

### Mediation Through Perinatal Factors

Preterm birth and low birth weight were associated with ART conception and non-ART conception (eTable 4 and 5 in the Supplement). They were also associated with increased risks of any type of childhood cancers and hepatic tumors, but not leukemias (eTable 6 in the Supplement). Thus, preterm birth and low birth weight met the criteria for potential mediators. However, mediation analyses showed that they did not mediate the association between ART conception and any type of childhood cancer and hepatic tumors (Table 4).

**Table 4.** Mediation Analysis for the Association Between ART Conception and Childhood Cancers With Preterm Birth or Low Birth Weight as a Potential Mediator^a^

4-Way decompositions of total association	Estimate (95% CI)			
	Any type of cancer		Hepatic tumors	
	Reference, natural conception	Reference, non-ART conception	Reference, natural conception	Reference, non-ART conception
**Preterm birth (<37 wk)**				
Controlled direct association	0.57 (−0.24 to 1.38)	3.21 (−0.83 to 7.24)	0.52 (−0.26 to 1.29)	0.97 (−3.05 to 5.00)
Reference interaction	−0.16 (−1.06 to 0.74)	−2.28 (−6.35 to 1.78)	−0.21 (−1.03 to 0.62)	−0.18 (−4.01 to 3.64)
Mediated interaction	0.06 (−0.25 to 0.37)	0.79 (−0.62 to 2.20)	0.07 (−0.20 to 0.34)	0.06 (−1.20 to 1.32)
Pure indirect association	0.11 (−0.03 to 0.18)	0.04 (−0.22 to 0.29)	0.05 (−0.05 to 0.16)	0.61 (−0.09 to 1.12)
**Low birth weight (<2500 g)**				
Controlled direct association	0.78 (−0.05 to 1.60)	2.92 (−1.28 to 7.11)	0.35 (−0.46 to 1.16)	0.86 (−3.29 to 5.02)
Reference interaction	−0.36 (−1.28 to 0.56)	−2.06 (−6.26 to 2.15)	−0.05 (−0.91 to 0.82)	−0.13 (−4.11 to 3.85)
Mediated interaction	0.13 (−0.19 to 0.45)	0.72 (−0.75 to 2.18)	0.02 (−0.27 to 0.30)	0.04 (−1.25 to 1.33)
Pure indirect association	0.05 (−0.03 to 0.12)	0.18 (−0.15 to 0.51)	0.11 (−0.01 to 0.23)	0.65 (−0.07 to 1.23)

Abbreviation: ART, assisted reproductive technology.

^a^ Estimates were reported as excess relative risks after adjusting for maternal age, paternal age, child’s birth year, child’s sex, parity, socioeconomic status, residential urbanization level, and abortion history.

## Discussion

In this cohort study using nationwide data from Taiwan between 2004 and 2017, ART conception was associated with an increased risk of childhood cancers when compared with natural conception or subfertility and non-ART conception. The increased childhood cancer risk was mainly due to the occurrence of leukemias and hepatic tumors. The risk of childhood cancers was not significantly different when comparing subfertility and non-ART conception with natural conception. This study also showed that the observed associations were not mediated by preterm birth and low birth weight. Our findings suggest that couples seeking ART treatment need to be informed of the low but significantly increased risk of childhood cancers. Health care workers should collect information on the mode of conception and screen children conceived through ART for early detection of childhood cancers.

Only 4 nationwide studies, generally comparable to this study, examined the associations of ART conception and natural conception with overall childhood cancers.^16,17,21,22^ Two of these studies reported similar results to that of our study, ie, a significant association between ART conception and an increased risk of overall childhood cancers compared with natural conception.^16,17^ The results of the other 2 studies were in contrast to our findings. A Danish cohort study with a mean follow-up of 11.3 years and a Dutch study using records of ART clinics with a median follow-up of 21 years found no statistically significant association between ART conception and overall childhood cancers after adjusting only for the year of birth^21^ or the parental cause of infertility,^22^ respectively. These studies had some critical limitations: a lack of infertility diagnosis and the amount of missing data on the conception method.^21,22^ These limitations may have led to nondifferential misclassifications and bias toward the null.

Few studies have examined the association between ART conception and specific types of cancers. A US study using administrative data from 14 states with a mean follow-up of 4.6 years^17^ reported an increased risk of hepatic tumors in children born after ART conception compared with non-ART conception, which concurred with our findings. A Nordic population-based cohort study reported ART conception being associated with a 2.61-times higher risk of hepatic tumors than natural conception,^51^ similar to the risk estimates of our study. However, the Nordic study did not find a significant difference (95% CI, 0.74-9.26), which may be attributed to the limited number of cases in their study.

Furthermore, we found that children born after ART conception were at an increased risk of leukemias compared with those born after natural conception, consistent with results of a study using nationwide data from Greece and Sweden between 1995 and 2008 (OR, 2.21; 95% CI, 1.27-3.85).^15^ However, a US study observed no such association with leukemia,^17^ possibly owing to the shorter follow-up time (mean, 4.6 years). A Danish study found a 2.87-fold increased risk of leukemia (95% CI, 1.19-6.93) compared with natural conception only for children conceived using frozen embryo transfer.^21^ However, in our study, no association between the use of frozen embryos and cancers was found.

This study found significant differences in the risk of childhood cancers when comparing ART conception with subfertility and non-ART conception, but no significant differences were found between natural conception and subfertility with no use of ART. Those comparisons suggest that the increased cancer risk may be due to ART treatment rather than subfertility. However, ART conception may be a proxy for more severe infertility.

Although preterm birth and low birth weight seem to be plausible mechanisms,^6,7,8,11,12,13,14^ the findings of this study suggest that these variables did not mediate the association between ART conception and childhood cancers. However, we found that the other decomposed associations were not significant either, suggesting that the limited number of cases may have influenced the results.

### Strengths and Limitations

This study has several strengths. The long-term and nationwide registry data were used to reduce the possibility of selection bias, loss to follow-up, and recall bias. This study is, to our knowledge, the first and largest investigation into an Asian population. This study also controlled and adjusted for several potential confounders, unlike previous studies. Additionally, 3 groups of exposure comparisons provided a more complete understanding of the association between the mode of conception and childhood cancers. Also, this is the first study to offer a plausible mechanism for the association through perinatal outcomes using mediation analysis.

Our study has some limitations. First, although this study used data from the entire population in Taiwan, Taiwan’s lower incidence rate of childhood cancers resulted in a limited number of cases for various types of childhood cancers and consequently a failure to accurately analyze them.^52^ Second, we could not rule out the possibility of a failure to identify the mediating role of preterm births and low birth weight owing to the limited number of cases in the mediation analysis. Third, this study did not have data on intracytoplasmic sperm injection. Further division of the ART group was not possible. Additionally, the lack of information on out-migration might lead to misclassification. The possibility of unknown and residual confounding could not be ruled out; however, it required fairly substantial confounding to explain away. We were not able to verify sequential ignorability assumptions in the interpretation of mediation analyses, but we have employed covariate adjustment in our analyses to make the assumptions plausible.

## Conclusions

This cohort study showed that regardless of comparison to natural conception or subfertility with no use of ART, ART conception was associated with increased risks for any type of childhood cancers, leukemias, and hepatic tumors. These associations were not mediated by preterm birth and low birth weight. Despite the reported increased risk of childhood cancers, the incidence rate remains low.

## Notes

Supplement. **eAppendix. **Details on the seven national administrative databases **eFigure 1. **The process of linking the seven national administrative databases **eFigure 2.** Criteria for children who were conceived through ART **eFigure 3. **Flow diagram of study participants **eTable 1.** Classification table from the ICCC-3 **eTable 2. **Association between childhood cancers and mode of conception stratified by the source of infertility and embryo type **eTable 3.** Risk of childhood cancers by mode of conception for boys and girls **eTable 4.** Models for mediators and mode of conception as independent variables **eTable 5.** Models for mediators and ART conception as independent variables compared with subfertility and non-ART conception **eTable 6.** Models for childhood cancers and mediators as independent variables
eReferences

## References

[1] de Mouzon J, Chambers GM, Zegers-Hochschild F, . International Committee for Monitoring Assisted Reproductive Technologies world report: assisted reproductive technology 2012. Hum Reprod. 2020;35(8):1900-1913. doi:10.1093/humrep/deaa090 32699900

[2] Department of Household Registration. Statistics for number of births. Department of Household Registration, Ministry of Interior. Accessed September 9, 2021. https://www.ris.gov.tw/app/portal/346

[3] Health Promotion Administration MoHaW. The Assisted Reproductive Technology Summary 2018 National Report of Taiwan. 2020. Accessed June 24, 2022. https://www.hpa.gov.tw/File/Attach/12907/File_14922.pdf

[4] Mani S, Ghosh J, Coutifaris C, Sapienza C, Mainigi M. Epigenetic changes and assisted reproductive technologies. Epigenetics. 2020;15(1-2):12-25. doi:10.1080/15592294.2019.1646572 31328632 PMC6961665

[5] Huntriss J, Balen AH, Sinclair KD, Brison DR, Picton HM; Royal College of Obstetricians Gynaecologists. Epigenetics and reproductive medicine: scientific impact paper No. 57. BJOG. 2018;125(13):e43-e54. doi:10.1111/1471-0528.15240 30251316

[6] Jackson RA, Gibson KA, Wu YW, Croughan MS. Perinatal outcomes in singletons following in vitro fertilization: a meta-analysis. Obstet Gynecol. 2004;103(3):551-563. doi:10.1097/01.AOG.0000114989.84822.51 14990421

[7] Marino JL, Moore VM, Willson KJ, . Perinatal outcomes by mode of assisted conception and sub-fertility in an Australian data linkage cohort. PLoS One. 2014;9(1):e80398. doi:10.1371/journal.pone.0080398 24416127 PMC3885393

[8] Qin J, Liu X, Sheng X, Wang H, Gao S. Assisted reproductive technology and the risk of pregnancy-related complications and adverse pregnancy outcomes in singleton pregnancies: a meta-analysis of cohort studies. Fertil Steril. 2016;105(1):73-85.e1-6. doi:10.1016/j.fertnstert.2015.09.007 26453266

[9] Benetatos L, Vartholomatos G. Imprinted genes in myeloid lineage commitment in normal and malignant hematopoiesis. Leukemia. 2015;29(6):1233-1242. doi:10.1038/leu.2015.47 25703588

[10] Finegold MJ, López-Terrada DH. Hepatic Tumors in Childhood. In: Russo P, Ruchelli ED, Piccoli DA, eds. Pathology of Pediatric Gastrointestinal and Liver Disease. Springer Berlin Heidelberg; 2014:547-614.

[11] Herzog CE, Andrassy RJ, Eftekhari F. Childhood cancers: hepatoblastoma. Oncologist. 2000;5(6):445-453. doi:10.1634/theoncologist.5-6-445 11110595

[12] Caughey RW, Michels KB. Birth weight and childhood leukemia: a meta-analysis and review of the current evidence. Int J Cancer. 2009;124(11):2658-2670. doi:10.1002/ijc.24225 19173295

[13] Paquette K, Coltin H, Boivin A, Amre D, Nuyt A-M, Luu TM. Cancer risk in children and young adults born preterm: A systematic review and meta-analysis. PLoS One. 2019;14(1):e0210366. doi:10.1371/journal.pone.0210366 30608983 PMC6319724

[14] Heck JE, Lee P-C, Wu C-K, . Gestational risk factors and childhood cancers: a cohort study in Taiwan. Int J Cancer. 2020;147(5):1343-1353. doi:10.1002/ijc.32905 32020595

[15] Petridou ET, Sergentanis TN, Panagopoulou P, . In vitro fertilization and risk of childhood leukemia in Greece and Sweden. Pediatr Blood Cancer. 2012;58(6):930-936. doi:10.1002/pbc.23194 21618418

[16] Wainstock T, Walfisch A, Shoham-Vardi I, . Fertility treatments and pediatric neoplasms of the offspring: results of a population-based cohort with a median follow-up of 10 years. Am J Obstet Gynecol. 2017;216(3):314.e1-314.e14. doi:10.1016/j.ajog.2017.01.015 28153657

[17] Spector LG, Brown MB, Wantman E, . Association of in vitro fertilization with childhood cancer in the United States. JAMA Pediatr. 2019;173(6):e190392. doi:10.1001/jamapediatrics.2019.0392 30933244 PMC6547076

[18] Williams CL, Bunch KJ, Stiller CA, . Cancer risk among children born after assisted conception. N Engl J Med. 2013;369(19):1819-1827. doi:10.1056/NEJMoa1301675 24195549

[19] Wang T, Chen L, Yang T, . Cancer risk among children conceived by fertility treatment. Int J Cancer. 2019;144(12):3001-3013. doi:10.1002/ijc.32062 30548591 PMC6590158

[20] Rudant J, Amigou A, Orsi L, . Fertility treatments, congenital malformations, fetal loss, and childhood acute leukemia: the ESCALE study (SFCE). Pediatr Blood Cancer. 2013;60(2):301-308. doi:10.1002/pbc.24192 22610722

[21] Hargreave M, Jensen A, Hansen MK, . Association between fertility treatment and cancer risk in children. JAMA. 2019;322(22):2203-2210. doi:10.1001/jama.2019.18037 31821431 PMC7081748

[22] Spaan M, van den Belt-Dusebout AW, van den Heuvel-Eibrink MM, ; OMEGA-steering group. Risk of cancer in children and young adults conceived by assisted reproductive technology. Hum Reprod. 2019;34(4):740-750. doi:10.1093/humrep/dey394 30715305 PMC6443110

[23] Zhang Y, Gao R, Chen H, . The association between fertility treatments and the incidence of paediatric cancer: a systematic review and meta-analysis. Eur J Cancer. 2020;138:133-148. doi:10.1016/j.ejca.2020.08.001 32889368

[24] American College of Obstetricians and Gynecologists’ Committee on Obstetric Practice; Committee on Genetics; US Food and Drug Administration. Committee opinion No 671: perinatal risks associated with assisted reproductive technology. Obstet Gynecol. 2016;128(3):e61-e68. doi:10.1097/AOG.0000000000001643 27548556

[25] Li C-Y, Chen LH, Chiou MJ, Liang FW, Lu T-H. Set-up and future applications of the Taiwan Maternal and Child Health Database (TMCHD). Taiwan J Public Health. 2016;35(2):209-220. doi:10.6288/tjph201635104053

[26] Lin CM, Lee PC, Teng SW, Lu TH, Mao IF, Li CY. Validation of the Taiwan Birth Registry using obstetric records. J Formos Med Assoc. 2004;103(4):297-301. 15175826

[27] Chiang CJ, You SL, Chen CJ, Yang YW, Lo WC, Lai MS. Quality assessment and improvement of nationwide cancer registration system in Taiwan: a review. Jpn J Clin Oncol. 2015;45(3):291-296. doi:10.1093/jjco/hyu211 25601947

[28] Lu TH, Lee MC, Chou MC. Accuracy of cause-of-death coding in Taiwan: types of miscoding and effects on mortality statistics. Int J Epidemiol. 2000;29(2):336-343. doi:10.1093/ije/29.2.336 10817134

[29] Hsieh CY, Su CC, Shao SC, . Taiwan’s National Health Insurance Research Database: past and future. Clin Epidemiol. 2019;11:349-358. doi:10.2147/CLEP.S196293 31118821 PMC6509937

[30] Ministry of Health and Welfare. Assisted Reproduction Act. Accessed February 18, 2021. https://law.moj.gov.tw/ENG/LawClass/LawAll.aspx?pcode=L0070024

[31] Steliarova-Foucher E, Stiller C, Lacour B, Kaatsch P. International Classification of Childhood Cancer, third edition. Cancer. 2005;103(7):1457-1467. doi:10.1002/cncr.20910 15712273

[32] Wang R, Metayer C, Morimoto L, . Parental age and risk of pediatric cancer in the offspring: a population-based record-linkage study in California. Am J Epidemiol. 2017;186(7):843-856. doi:10.1093/aje/kwx160 28535175 PMC5860074

[33] Balasch J. Ageing and infertility: an overview. Gynecol Endocrinol. 2010;26(12):855-860. doi:10.3109/09513590.2010.501889 20642380

[34] Brandt JS, Cruz Ithier MA, Rosen T, Ashkinadze E. Advanced paternal age, infertility, and reproductive risks: a review of the literature. Prenat Diagn. 2019;39(2):81-87. doi:10.1002/pd.5402 30520056

[35] Alvarez S. Do some addictions interfere with fertility? Fertil Steril. 2015;103(1):22-26. doi:10.1016/j.fertnstert.2014.11.008 25552409

[36] Frederiksen LE, Erdmann F, Wesseling C, Winther JF, Mora AM. Parental tobacco smoking and risk of childhood leukemia in Costa Rica: a population-based case-control study. Env Res. 2020;180:108827. doi:10.1016/j.envres.2019.108827 31655332

[37] Auger N, Goudie C, Low N, Healy-Profitós J, Lo E, Luu TM. Maternal use of illicit drugs, tobacco or alcohol and the risk of childhood cancer before 6 years of age. Drug Alc Dep. 2019;200:133-138. doi:10.1016/j.drugalcdep.2019.03.008 31129483

[38] Männistö J, Mentula M, Bloigu A, Gissler M, Heikinheimo O, Niinimäki M. Induced abortion and future use of IVF treatment; A nationwide register study. PLoS One. 2019;14(11):e0225162. doi:10.1371/journal.pone.0225162 31725766 PMC6855489

[39] Parodi S, Merlo DF, Ranucci A, . Risk of neuroblastoma, maternal characteristics and perinatal exposures: the SETIL study. Cancer Epidemiol. 2014;38(6):686-694. doi:10.1016/j.canep.2014.09.007 25280392

[40] Kehm RD, Spector LG, Poynter JN, Vock DM, Osypuk TL. Socioeconomic status and childhood cancer incidence: a population-based multilevel analysis. Am J Epidemiol. 2018;187(5):982-991. doi:10.1093/aje/kwx322 29036606 PMC5928452

[41] Li CY, Lin RS, Lin CH. Urbanization and childhood leukaemia in Taiwan. Int J Epidemiol. 1998;27(4):587-591. doi:10.1093/ije/27.4.587 9758111

[42] National Health Insurance Administration MoHaW. How Premiums Are Calculated. National Health Insurance Administration. Accessed March 5, 2020. https://www.nhi.gov.tw/English/Content_List.aspx?n=B9C9C690524F2543&topn=46FA76EB55BC2CB8

[43] Liu CHY, Chuang Y, Chen Y, . Incorporating development stratification of Taiwan townships into sampling design of large scale health interview survey. J Health Manag. 2006;4:1-22. doi:10.29805/JHM.200606.0001

[44] Valeri L, VanderWeele TJ. Mediation analysis allowing for exposure-mediator interactions and causal interpretation: theoretical assumptions and implementation with SAS and SPSS macros. Psychol Methods. 2013;18(2):137-150. doi:10.1037/a0031034 23379553 PMC3659198

[45] Jakobsen JC, Gluud C, Wetterslev J, Winkel P. When and how should multiple imputation be used for handling missing data in randomised clinical trials—a practical guide with flowcharts. BMC Med Res Methodol. 2017;17(1):162. doi:10.1186/s12874-017-0442-1 29207961 PMC5717805

[46] StataCorp LLC. Stata multiple-imputation reference manual. 2021. https://www.stata.com/manuals/mi.pdf

[47] VanderWeele TJ, Ding P. Sensitivity analysis in observational research: introducing the e-value. Ann Intern Med. 2017;167(4):268-274. doi:10.7326/M16-2607 28693043

[48] Discacciati A, Bellavia A, Lee JJ, Mazumdar M, Valeri L. Med4way: a Stata command to investigate mediating and interactive mechanisms using the four-way effect decomposition. Int J Epidemiol. 2018. doi:10.1093/ije/dyy236 30452641

[49] VanderWeele TJ. A unification of mediation and interaction: a 4-way decomposition. Epidemiology. 2014;25(5):749-761. doi:10.1097/EDE.0000000000000121 25000145 PMC4220271

[50] StataCorp L. Stata Statistical Software: Release 15 (2017). StataCorp LP; 2017.

[51] Sundh KJ, Henningsen A-KA, Källen K, . Cancer in children and young adults born after assisted reproductive technology: a Nordic cohort study from the Committee of Nordic ART and Safety (CoNARTaS). Hum Reprod. 2014;29(9):2050-2057. doi:10.1093/humrep/deu143 24990274

[52] Liu YL, Lo WC, Chiang CJ, . Incidence of cancer in children aged 0-14 years in Taiwan, 1996-2010. Cancer Epidemiol. 2015;39(1):21-28. doi:10.1016/j.canep.2014.11.010 25599927

