# Supplementary material for: Assisted Reproductive Technology and Risk of Childhood Cancers
Source: JAMA Netw Open. 2022 Aug 31;5(8):e2230157. doi: 10.1001/jamanetworkopen.2022.30157 (PMC9434352; doi:10.1001/jamanetworkopen.2022.30157)
Supplement: Supplement. — eAppendix. Details on the seven national administrative databases eFigure 1. The process of linking the seven national administrative databases eFigure 2. Criteria for children who were conceived through ART eFigure 3. Flow diagram of study participants eTable 1. Classification table from the ICCC-3 eTable 2. Association between childhood cancers and mode of conception stratified by the source of infertility and embryo type eTable 3. Risk of childhood cancers by mode of conception for boys and girls eTable 4. Models for mediators and mode of conception as independent variables eTable 5. Models for mediators and ART conception as independent variables compared with subfertility and non-ART conception eTable 6. Models for childhood cancers and mediators as independent variables eReferences [file jamanetwopen-e2230157-s001.pdf]

Supplementary Online Content

Weng SS, Huang YT, Huang YT, Li YP, Chien LY. Assisted reproductive technology and risk of childhood cancers. *JAMA Netw Open*. 2022;5(8):e2230157. doi:10.1001/jamanetworkopen.2022.30157

eAppendix. Details on the seven national administrative databases
eFigure 1. The process of linking the seven national administrative databases
eFigure 2. Criteria for children who were conceived through ART
eFigure 3. Flow diagram of study participants
eTable 1. Classification table from the ICCC-3
eTable 2. Association between childhood cancers and mode of conception stratified by the source of infertility and embryo type
eTable 3. Risk of childhood cancers by mode of conception for boys and girls
eTable 4. Models for mediators and mode of conception as independent variables
eTable 5. Models for mediators and ART conception as independent variables compared with subfertility and non-ART conception
eTable 6. Models for childhood cancers and mediators as independent variables
eReferences

This supplemental material has been provided by the authors to give readers additional information about their work.

© 2022 Weng SS et al. *JAMA Network Open.*

eAppendix. Details on the seven national administrative databases

The ***Maternal and Child Health Database*** collects administrative data that can correctly identify the parents of an offspring and contains 99.78% of all parent-child pairs in Taiwan with their unique identifiers.[1] The ***Artificial Reproductive Data*** registry is a compulsory national database.[2] It was established based on legal requirements regulating that each assisted reproduction institution in Taiwan reports for all cases the information on assisted reproductive technology (ART) treatment, such as ART methods, causes of infertility, types of embryos, and clinical pregnancy.[3]

The databases of the ***Registry for Beneficiaries*** and ***Ambulatory Care Expenditures by Visits*** are claims databases of the National Health Insurance (NHI) program, which is a compulsory social insurance that covers 99.6% of Taiwan's residents.[4] These databases manage information such as household income basis (NT$), medical diagnoses coded using the International Classification of Diseases, Ninth Revision, Clinical Modification (ICD-9-CM) before 30 September 2015 and ICD-10-CM afterwards, as well as the date of diagnosis. The validity of the diagnosis codes has modest-to-high sensitivity and positive predictive values.[5]

The ***Birth Certificate Application*** registry is regulated by the Protection of Children and Youth Welfare and Rights Act ensuring that medical facilities report relevant birth information within seven days of birth. Therefore, it contains data on all births and includes information on the date of birth, sex of the child, gestational age, birth weight, parent's original nationality, parent's age, mother's residential township at delivery, parity, multiple gestations, and pregnancy risk behaviors (including tobacco, drinking, and substance addiction).[6] The obstetric records in the Birth Certificate Application have been evaluated as valid and complete.[7]

The ***Taiwan Cancer Registry Data*** registry contains data on all incident cases of cancers, such as the age at diagnosis and the morphology and topography codes according to the International Classification of Diseases for Oncology (ICD-O). Hospitals are required to report cancer information based on the Cancer Control Act 2003. The completeness of this database (97%) and its data quality

© 2022 Weng SS et al. *JAMA Network Open.*

make it an excellent resource.[8] The ***Cause of Death Data*** registry records all deaths for Taiwanese citizens including the age at death, and its accuracy and completeness have been documented.[9]

Before requesting access to these national administrative databases, researchers must receive approval from the IRB. Additionally, peer experts review and approve the use of these databases to ensure the appropriateness of their use. All national administrative databases are managed and stored by the Health and Welfare Data Centre, Ministry of Health and Welfare. The names of the included individuals are encrypted and anonymized using unique identifiers. To protect personal information, researchers are required to conduct on-site analyses at the Health and Welfare Data Center. All data intended to be brought out from this center must be reviewed to prevent the possibility of disclosing an individual's identity.

© 2022 Weng SS et al. *JAMA Network Open.*

eFigure 1. The process of linking the seven national administrative databases

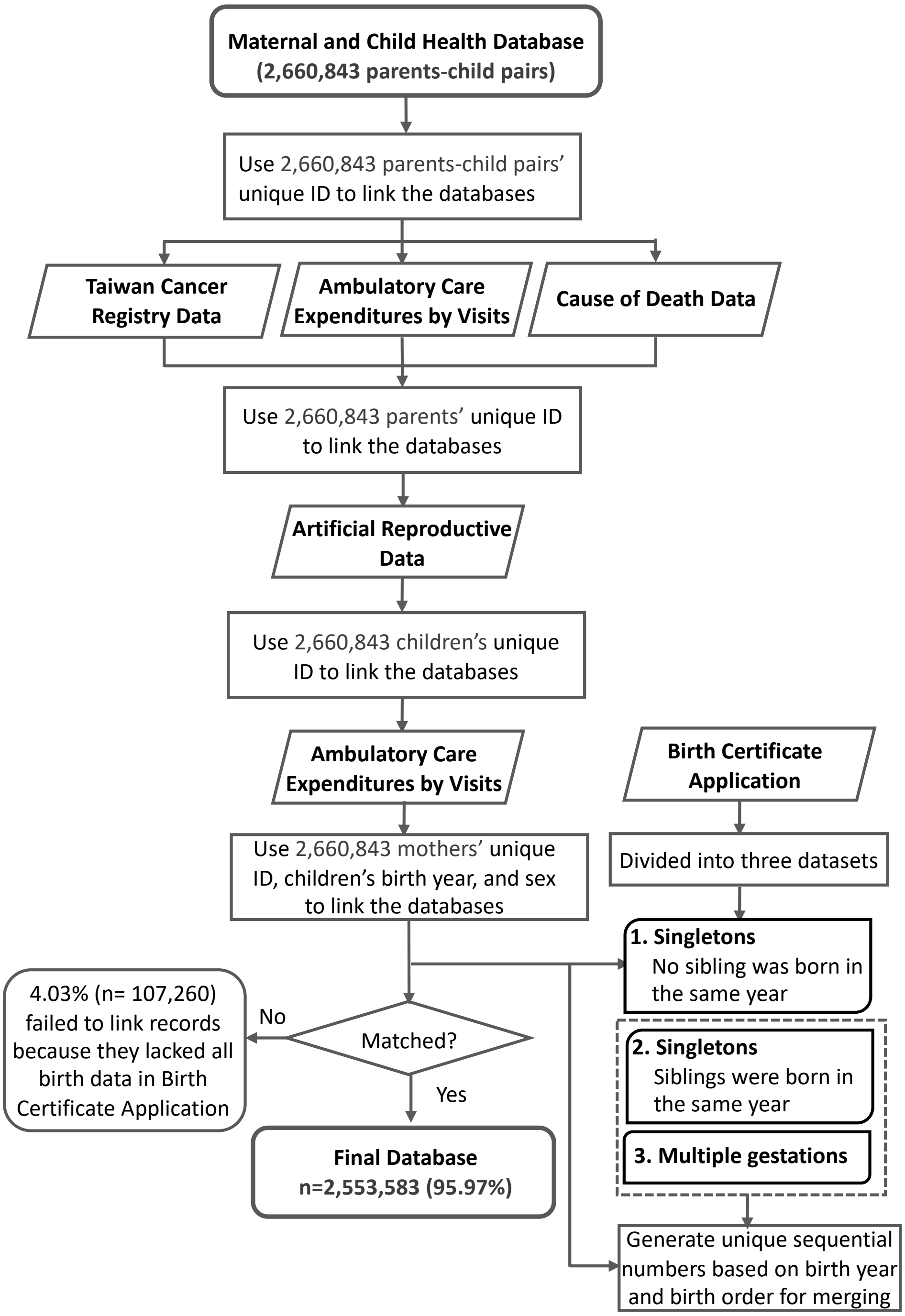

Note. All individuals were assigned a unique personal identification (ID) number in the national administrative databases. This enables linkage to other national administrative databases. The unique ID numbers in the Maternal and Child Health Database allow the identification of an individual's parents and siblings. Therefore, the ID number of the Maternal and Child Health Database was used to link the following six national administrative databases: Artificial Reproductive Data, Registry for Beneficiaries, Ambulatory Care Expenditures by Visits, Birth Certificate Application, Taiwan Cancer Registry Data, and Cause of Death Data. The Artificial Reproductive Data database did not have a child's ID. Hence, to identify which child in a household was born after ART, we required that the offspring's birth date be within 290 days following the date of transfer, after using the parents' ID to link to the Registry for Beneficiaries database. Then, because the Birth Certificate Application database only contains mothers' IDs, it cannot identify birth data if a mother gave birth twice in the same year or had multiple gestations.

© 2022 Weng SS et al. *JAMA Network Open.*

Therefore, the Birth Certificate Application database was divided into three datasets: (1) all singletons and no siblings born in the same year, (2) siblings born in the same year, and (3) multiple gestations. The variables for the linkage between the first dataset–(all singletons and no siblings born in the same year) and the merged Maternal and Child Health Database were mothers' ID, children's birth year, and sex. As for the other two datasets, siblings born in the same year and multiple gestations, an additional linkage variable, i.e., a unique sequential number generated for each mother's birth data based on birth year and birth order, was used. After linking the seven national administrative databases, 95.97% of the parents-child pairs (n=2,553,583) were matched successfully. There were 4.03% of parents-child pairs (n=107,260) who failed to be linked to the Birth Certificate Application records, and most of them lacked all birth data.

© 2022 Weng SS et al. *JAMA Network Open.*

eFigure 2. Criteria for children who were conceived through ART

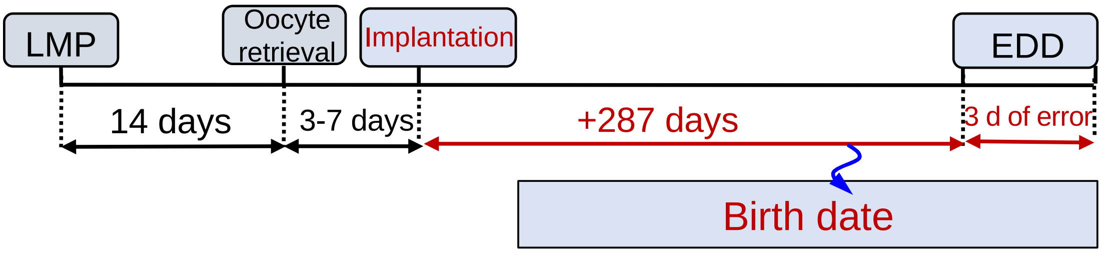

Abbreviations: ART, assisted reproductive technology; LMP, last menstrual period; EDD, estimated delivery date.

To minimize possible misclassifications, children conceived through ART were defined as those for whom one of the parents had an infertility diagnosis, a clinical pregnancy was recorded after ART, and the birth date was within 290 days after the transfer date. The 290 days were derived by subtracting from 308 days (44 weeks of gestational age) 14 days (the duration between LMP and retrievals), subtracting another 7 days (to account for 3–7 days in embryo culture), and adding 3 days of errors. A previous study indicated that the mean difference in gestational age was 0.9–2.1 days between the methods using the last menstrual period, crown-rump length, and biparietal diameter; therefore, an error of 3 days was used.[10]

© 2022 Weng SS et al. *JAMA Network Open.*

eFigure 3. Flow diagram of study participants

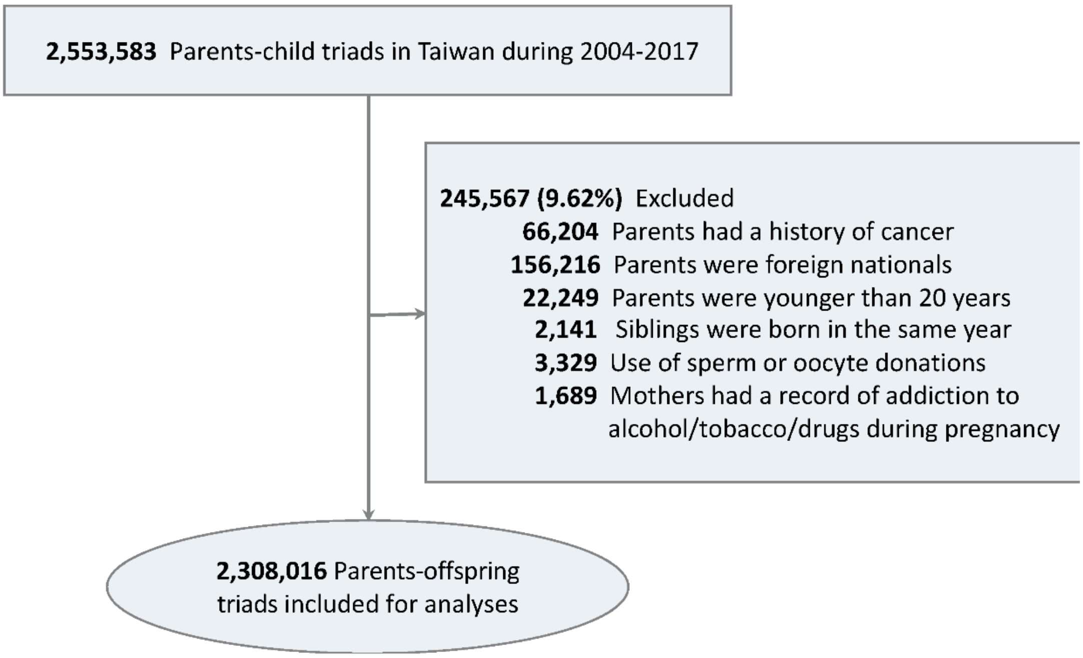

© 2022 Weng SS et al. *JAMA Network Open.*

eTable 1. Classification table from the ICCC-3[11]

**Cancers groups**	**ICD-O-3 morphology**	**ICD-O-3 topography**
I. Leukemias		
(a) Lymphoid leukemias	9820, 9823, 9826, 9827, 9831-9837, 9940, 9948	C000-C809
(b) Acute myeloid leukemias	9840, 9861, 9866, 9867, 9870-9874, 9891, 9895-9897, 9910, 9920, 9931	C000-C809
(c) Chronic myeloproliferative diseases	9863, 9875, 9876, 9950, 9960-9964	C000-C809
(d) Myelodysplastic syndrome and other myeloproliferative diseases	9945, 9946, 9975, 9980, 9982-9987, 9989	C000-C809
(e) Unspecified and other specified leukemias	9800, 9801, 9805, 9860, 9930	C000-C809
II. Lymphomas and reticuloendothelial neoplasms		
(a) Hodgkin lymphomas	9650-9655, 9659, 9661-9665, 9667	C000-C809
(b) Non-Hodgkin lymphomas (except Burkitt lymphoma)	9591, 9670, 9671, 9673, 9675, 9678-9680, 9684, 9689-9691, 9695, 9698-9702, 9705, 9708, 9709, 9714, 9716-9719, 9727-9729, 9731-9734, 9760-9762, 9764-9769, 9970	C000-C809
(c) Burkitt lymphoma	9687	C000-C809
(d) Miscellaneous lymphoreticular neoplasms	9740-9742, 9750, 9754-9758	C000-C809
(e) Unspecified lymphomas	9590, 9596	C000-C809
III. CNS and miscellaneous intracranial and intraspinal neoplasms		
(a) Ependymomas	9383, 9390-9394	C000-C809
(b) Astrocytomas	9380	C723
	9384, 9400-9411, 9420, 9421-9424, 9440-9442	C000-C809
(c) Intracranial and intraspinal embryonal tumors	9470-9474, 9480, 9508	C000-C809
	9501-9504	C700-C729
(d) Other gliomas	9380	C700-C722, C724-C729, C751, C753
	9381, 9382, 9430, 9444, 9450, 9451, 9460	C000-C809

© 2022 Weng SS et al. *JAMA Network Open.*

(e) Other specified intracranial and intraspinal neoplasms	8270-8281, 8300, 9350-9352, 9360-9362, 9412, 9413, 9492, 9493, 9505-9507, 9530-9539, 9582	C000-C809
(f) Unspecified intracranial and intraspinal neoplasms	8000-8005	C700-C729, C751-C753
IV. Neuroblastoma and other peripheral nervous cell tumors		
(a) Neuroblastoma and ganglioneuroblastoma	9490, 9500	C000-C809
(b) Other peripheral nervous cell tumors	8680-8683, 8690-8693, 8700, 9520-9523	C000-C809
	9501-9504	C000-C699, C739-C768, C809
V. Retinoblastoma	9510-9514	C000-C809
VI. Renal tumors		
(a) Nephroblastoma and other nonepithelial renal tumors	8959, 8960, 8964-8967	C000-C809
	8963, 9364	C649
(b) Renal carcinomas	8010-8041, 8050-8075, 8082, 8120-8122, 8130-8141, 8143, 8155, 8190-8201, 8210, 8211, 8221-8231, 8240, 8241, 8244-8246, 8260-8263, 8290, 8310, 8320, 8323, 8401, 8430, 8440, 8480-8490, 8504, 8510, 8550, 8560-8576	C649
	8311, 8312, 8316-8319, 8361	C000-C809
(c) Unspecified malignant renal tumors	8000-8005	C649
VII. Hepatic tumors		
(a) Hepatoblastoma	8970	C000-C809
(b) Hepatic carcinomas	8010-8041, 8050-8075, 8082, 8120-8122, 8140, 8141, 8143, 8155, 8190-8201, 8210, 8211, 8230, 8231, 8240, 8241, 8244-8246, 8260-	C220, C221

© 2022 Weng SS et al. *JAMA Network Open.*

	8264, 8310, 8320, 8323, 8401, 8430, 8440, 8480-8490, 8504, 8510, 8550, 8560-8576	
	8160-8180	C000-C809
(c) Unspecified malignant hepatic tumors	8000-8005	C220, C221
VIII. Malignant bone tumors		
(a) Osteosarcomas	9180-9187, 9191-9195, 9200	C400-C419, C760-C768, C809
(b) Chondrosarcomas	9210, 9220, 9240	C400-C419, C760-C768, C809
	9221, 9230, 9241-9243	C000-C809
(c) Ewing tumor and related sarcomas of bone	9260	C400-C419, C760-C768, C809
	9363-9365	C400-C419
(d) Other specified malignant bone tumors	8810, 8811, 8823, 8830	C400-C419
	8812, 9250, 9261, 9262, 9270-9275, 9280-9282, 9290, 9300-9302, 9310-9312, 9320-9322, 9330, 9340-9342, 9370-9372	C000-C809
(e) Unspecified malignant bone tumors	8000-8005, 8800, 8801, 8803-8805	C400-C419
IX. Soft tissue and other extraosseous sarcomas		
(a) Rhabdomyosarcomas	8900-8905, 8910, 8912, 8920, 8991	C000-C809
(b) Fibrosarcomas, peripheral nerve sheath tumors, and other fibrous neoplasms	8810, 8811, 8813-8815, 8821, 8823, 8834-8835	C000-C399, C440-C768, C809
	8820, 8822, 8824-8827, 9150, 9160, 9491, 9540-9571, 9580	C000-C809
(c) Kaposi sarcoma	9140	C000-C809

© 2022 Weng SS et al. *JAMA Network Open.*

(d) Other specified soft tissue sarcomas	8587, 8710-8713, 8806, 8831-8833, 8836, 8840-8842, 8850-8858, 8860-8862, 8870, 8880, 8881, 8890-8898, 8921, 8982, 8990, 9040-9044, 9120-9125, 9130-9133, 9135, 9136, 9141, 9142, 9161, 9170-9175, 9231, 9251, 9252, 9373, 9581	C000-C809
	8830	C000-C399, C440-C768, C809
	8963	C000-C639, C659-C699, C739-C768, C809
	9180, 9210, 9220, 9240	C490-C499
	9260	C000-C399, C470-C759
	9364	C000-C399, C470-C639, C659-C699, C739-C768, C809
	9365	C000-C399, C470-C639, C659-C768, C809
(e) Unspecified soft tissue sarcomas	8800-8805	C000-C399, C440-C768, C809
(e) Unspecified malignant bone tumors	8000-8005, 8800, 8801, 8803-8805	C400-C419
X. Germ cell tumors, trophoblastic tumors, and neoplasms of gonads		
(a) Intracranial and intraspinal germ cell tumors	9060-9065, 9070-9072, 9080-9085, 9100, 9101	C700-C729, C751-C753
(b) Malignant extracranial and extragonadal germ cell tumors	9060-9065, 9070-9072, 9080-9085, 9100-9105	C000-C559, C570-C619, C630-C699, C739-C750,

© 2022 Weng SS et al. *JAMA Network Open.*

		C754-C768, C809
(c) Malignant gonadal germ cell tumors	9060-9065, 9070-9073, 9080-9085, 9090, 9091, 9100, 9101	C569, C620-C629
(d) Gonadal carcinomas	8010-8041, 8050-8075, 8082, 8120-8122, 8130-8141, 8143, 8190-8201, 8210, 8211, 8221-8241, 8244-8246, 8260-8263, 8290, 8310, 8313, 8320, 8323, 8380-8384, 8430, 8440, 8480-8490, 8504, 8510, 8550, 8560-8573, 9000, 9014, 9015	C569, C620-C629
	8441-8444, 8450, 8451, 8460-8473	C000-C809
(e) Other and unspecified malignant gonadal tumors	8590-8671	C000-C809
XI. Other malignant epithelial neoplasms and malignant melanomas		
(a) Adrenocortical carcinomas	8370-8375	C000-C809
(b) Thyroid carcinomas	8010-8041, 8050-8075, 8082, 8120-8122, 8130-8141, 8190, 8200, 8201, 8211, 8230, 8231, 8244-8246, 8260-8263, 8290, 8310, 8320, 8323, 8430, 8440, 8480, 8481, 8510, 8560-8573	C739
	8330-8337, 8340-8347, 8350	C000-C809
(c) Nasopharyngeal carcinomas	8010-8041, 8050-8075, 8082, 8083, 8120-8122, 8130-8141, 8190, 8200, 8201, 8211, 8230, 8231, 8244-8246, 8260-8263, 8290, 8310, 8320, 8323, 8430, 8440, 8480, 8481, 8500-8576	C110-C119
(d) Malignant melanomas	8720-8780, 8790	C000-C809
(e) Skin carcinomas	8010-8041, 8050-8075, 8078, 8082, 8090-8110, 8140, 8143, 8147, 8190, 8200, 8240, 8246, 8247, 8260, 8310, 8320, 8323, 8390-8420, 8430, 8480, 8542, 8560, 8570-8573, 8940, 8941	C440-C449
(f) Other and unspecified carcinomas	8010-8084, 8120-8157, 8190-8264, 8290, 8310, 8313-8315, 8320-8325, 8360, 8380-8384, 8430-8440, 8452-8454, 8480-8586, 8588-8589, 8940, 8941, 8983, 9000, 9010-9016, 9020, 9030	C000-C109, C129-C218, C239-C399, C480-C488, C500-C559,

© 2022 Weng SS et al. *JAMA Network Open.*

		C570-C619, C630-C639, C659-C729, C750-C768, C809

© 2022 Weng SS et al. *JAMA Network Open.*

eTable 2. Association between childhood cancers and mode of conception stratified by the source of infertility and embryo type

Variables	All childhood cancers			Leukemias			Hepatic tumors		
	**No. of cases**	**Adjusted HR (95% CI)[a]**	**Adjusted HR (95% CI)[a]**	**No. of cases**	**Adjusted HR (95% CI)[a]**	**Adjusted HR (95% CI)[a]**	**No. of cases**	**Adjusted HR (95% CI)[a]**	**Adjusted HR (95% CI)[a]**
Natural conception	1,417	1 [Reference]	NA	348	1 [Reference]	NA	96	1 [Reference]	NA
Non-ART conception	416	NA	1 [Reference]	104	NA	1 [Reference]	30	NA	1 [Reference]
ART conception									
Source of infertility									
Paternal infertility	NR	3.36 (0.47 to 23.89)	2.99 (0.42 to 21.35)	0	NA	NA	0	NA	NA
Maternal infertility	34	1.71 (0.99 to 2.42)	1.59 (0.98 to 2.28)	6	1.43 (0.63 to 3.25)	1.35 (0.58 to 3.13)	NR	3.16 (0.86 to 7.45)	2.82 (0.97 to 7.12)
Both	NR	1.19 (0.67 to 2.11)	1.07 (0.60 to 1.92)	7	3.32 (0.76 to 7.08)	3.17 (0.75 to 6.94)	NR	1.90 (0.46 to 7.86)	1.69 (0.39 to 7.31)
Type of embryo									
Fresh	40	1.60 (1.20 to 2.27)	1.47 (1.06 to 2.05)	NR	1.99 (1.00 to 3.67)	1.79 (0.94 to 3.39)	8	2.71 (1.28 to 5.73)	2.41 (1.05 to 5.52)
Frozen	7	1.17 (0.55 to 2.47)	1.15 (0.54 to 2.46)	NR	2.42 (0.60 to 9.87)	2.30 (0.55 to 9.59)	0	NA	NA

Abbreviations: HR, hazard ratio; CI, confidence interval; NA, not applicable; NR, not reported due to the number being smaller than three to protect patient confidentiality under the Taiwan Data Protection Law.

[a] Adjusted hazard ratios and 95% confidence intervals were estimated from a Cox proportional hazard model adjusted for maternal age, paternal age, child's birth year, child's sex, parity, socioeconomic status, residential urbanization level, and abortion history.

© 2022 Weng SS et al. *JAMA Network Open.*

eTable 3. Risk of childhood cancers by mode of conception for boys and girls

Types of childhood cancers	Number of cases			HR (95% CI)[a]			HR (95% CI)[a]	
	Natural conception	Non-ART conception	ART conception	Natural conception	Non-ART conception	ART conception	Non-ART conception	ART conception
Boys								
Any type of cancer	806	232	25	1 [Reference]	1.16 (1.00 to 1.35)	1.49 (0.99 to 2.24)	1 [Reference]	1.33 (0.87 to 2.04)
Leukemia	205	63	6	1 [Reference]	1.27 (0.96 to 1.70)	1.67 (0.73 to 3.80)	1 [Reference]	1.37 (0.58 to 3.25)
Hepatic tumors	65	19	4	1 [Reference]	1.08 (0.64 to 1.81)	2.18 (0.77 to 6.17)	1 [Reference]	1.87 (0.60 to 5.84)
Girls								
Any type of cancer	611	184	22	1 [Reference]	1.11 (0.94 to 1.31)	1.68 (0.98 to 2.60)	1 [Reference]	1.54 (0.97 to 2.45)
Leukemia	143	41	7	1 [Reference]	1.09 (0.76 to 1.54)	2.75 (1.00 to 5.79)	1 [Reference]	2.77 (1.00 to 6.45)
Hepatic tumors	31	11	4	1 [Reference]	1.13 (0.56 to 2.26)	3.63 (0.99 to 10.78)	1 [Reference]	3.43 (0.99 to 11.64)

Abbreviations: HR, hazard ratio; CI, confidence interval; NA, not applicable due to no case or too few cases to estimate.

[a] Adjusted hazard ratios and 95% confidence intervals were estimated from a Cox proportional hazard model adjusted for maternal age, paternal age, child's birth year, child's sex, parity, socioeconomic status, residential urbanization level, and abortion history.

© 2022 Weng SS et al. *JAMA Network Open.*

eTable 4. Models for mediators and mode of conception as independent variables

Variables	Gestational age < 37 weeks[a]	Gestational age > 42 weeks[a]	Birth weight <2500g[b]	Birth weight >4200g[b]
	HR (95% CI)[d]	HR (95% CI)[d]	HR (95% CI)[d]	HR (95% CI)[d]
Natural conception	1 [Reference]	1 [Reference]	1 [Reference]	1 [Reference]
Subfertility and non-ART conception	1.23 (1.22 to 1.25)	0.66 (0.40 to 1.09)	1.22 (1.21 to 1.24)	1.04 (0.99 to 1.09)
ART conception	4.59 (4.49 to 4.69)	NA	5.36 (5.25 to 5.48)	0.47 (0.38 to 0.57)

Abbreviation: ART, assisted reproductive technology; HR, hazard ratios; CI, confidence interval; NA, not applicable due to no case.

[a] Reference group: 36-42 weeks of gestational age

[b] Reference group: 2500-4200 g of birth weight

[c] Reference group: singleton

[d] Adjusted for maternal age, paternal age, child's birth year, child's sex, parity, socioeconomic status, residential urbanization level, and abortion history.

© 2022 Weng SS et al. *JAMA Network Open.*

eTable 5. Models for mediators and ART conception as independent variables compared with subfertility and non-ART conception

Variables	Gestational age < 37 weeks[a]	Gestational age > 42 weeks[a]	Birth weight <2500g[b]	Birth weight >4200g[b]
	HR (95% CI)[d]	HR (95% CI)[d]	HR (95% CI)[d]	HR (95% CI)[d]
Subfertility and non-ART conception	1 [Reference]	1 [Reference]	1 [Reference]	1 [Reference]
ART conception	3.68 (3.59 to 3.77)	NA	4.32 (4.22 to 4.42)	0.46 (0.37 to 0.57)

Abbreviation: ART, assisted reproductive technology; HR, hazard ratios; CI, confidence interval; NA, not applicable due to no case.

[a] Reference group: 36-42 weeks of gestational age

[b] Reference group: 2500-4200 g of birth weight

[c] Reference group: singleton

[d] Adjusted for maternal age, paternal age, child's birth year, child's sex, parity, socioeconomic status, residential urbanization level, and abortion history.

© 2022 Weng SS et al. *JAMA Network Open.*

eTable 6. Models for childhood cancers and mediators as independent variables

Potential mediators	Any type of childhood cancer			Leukemias		Hepatic tumors	
	No. of children	No. of events	HR (95% CI)[a]	No. of events	HR (95% CI)[a]	No. of events	HR (95% CI)[a]
Gestational age							
< 37 weeks	188,231	210	1.31 (1.13 to 1.52)	51	1.29 (0.96 to 1.74)	24	1.83 (1.14 to 2.95)
37-42	1,967,502	1656	1 [Reference]	412	1 [Reference]	109	1 [Reference]
> 42 weeks	142	0	NA	0	NA	0	NA
Birth weight							
< 2500 g	170,312	178	1.23 (1.05 to 1.45)	38	1.10 (0.78 to 1.55)		2.20 (1.36 to 3.55)
2500-4200 g	1,974,629	1672	1 [Reference]	419	1 [Reference]	108	1 [Reference]
> 4200 g	10,934	16	1.58 (0.97 to 2.60)	6	2.26 (1.01 to 5.06)		1.40 (0.20 to 10.05)

Abbreviation: ART, assisted reproductive technology; HR, hazard ratios; CI, confidence interval; NA, not applicable due to no case.

[a] Adjusted for mode of conception, maternal age, paternal age, child's birth year, child's sex, parity, socioeconomic status, residential urbanization level, and abortion history.

© 2022 Weng SS et al. *JAMA Network Open.*

eReferences

1. Li CY, Chen LH, Chiou MJ, Liang FW, Lu TH. Set-up and future applications of the Taiwan Maternal and Child Health Database (TMCHD). *Taiwan Journal of Public Health* 2016;35:209-20. doi:10.6288/tjph201635104053
2. Wu CL, Rei W, Deng CY, Hsieh HY. National registries and health surveillance of assisted reproductive technologies: a comparative study. *Taiwan Journal of Public Health* 2017;36:6-20. doi:10.6288/tjph201736105099
3. Health Promotion Administration, Ministry of Health and Welfare. The Assisted Reproductive Technology Summary 2017 National Report of Taiwan. 2019. https://210.241.78.32/File/Attach/11584/File_13186.pdf
4. National Health Insurance Administration, Ministry of Health and Welfare. 2017-2018 National Health Insurance in Taiwan Annual Report (bilingual). 2020. https://www.nhi.gov.tw/English/Content_List.aspx?n=8FC0974BBFEFA56D&topn=ED4A30E51A609E49
5. Hsieh CY, Su CC, Shao SC, et al. Taiwan's National Health Insurance Research Database: past and future. *Clin Epidemiol* 2019;11:349-58. doi:10.2147/clep.S196293
6. Lin WH, Wang MC, Wang WM, et al. Incidence of and mortality from type I diabetes in Taiwan from 1999 through 2010: A nationwide cohort study. *PLoS One* 2014;9:e86172. doi:10.1371/journal.pone.0086172
7. Health Promotion Administration, Ministry of Health and Welfare. Annual report on birth notification. 2020. https://www.hpa.gov.tw/Pages/List.aspx?nodeid=55
8. Lin CM, Lee PC, Teng SW, Lu TH, Mao IF, Li CY. Validation of the Taiwan Birth Registry using obstetric records. *J Formos Med Assoc* 2004;103:297-301.
9. Chiang CJ, You SL, Chen CJ, Yang YW, Lo WC, Lai MS. Quality assessment and improvement of nationwide cancer registration system in Taiwan: a review. *Jpn J Clin Oncol* 2015;45:291-6. doi:10.1093/jjco/hyu211
10. Lu TH, Lee MC, Chou MC. Accuracy of cause-of-death coding in Taiwan: types of miscoding and effects on mortality statistics. *Int J Epidemiol* 2000;29:336-43. doi:10.1093/ije/29.2.336
11. Steliarova-Foucher E, Stiller C, Lacour B, Kaatsch P. International Classification of Childhood Cancer, third edition. *Cancer*. 3rd ed. 2005;103(7):1457-1467. doi: 10.1002/cncr.20910.

© 2022 Weng SS et al. *JAMA Network Open.*
